# A Rare Association of Takotsubo Cardiomyopathy with High-Degree Atrioventricular Block

**DOI:** 10.1155/2017/6989438

**Published:** 2017-09-20

**Authors:** Eder Hans Cativo, Rachna Valvani, Tuoyo O. Mene-Afejuku, Diana P. Cativo, Savi Mushiyev

**Affiliations:** ^1^Department of Internal Medicine, New York Medical College, Metropolitan Hospital Center, New York, NY, USA; ^2^Cardiology Division, Department of Internal Medicine, New York Medical College, Metropolitan Hospital Center, New York, NY, USA

## Abstract

Here we present a case of a patient who got trapped in an elevator; on initial evaluation patient was found with bradycardia; on further evaluation electrocardiogram (EKG) showed new onset 2nd-degree Mobitz type 2 AV block. On admission patient developed ischemic changes on EKG and troponin elevation. Transthoracic echocardiogram showed reduced ejection fraction as well as apical inferior, anterior, lateral, and septal hypokinesia. Coronary angiography showed nonobstructive coronary artery disease and ventriculogram demonstrated anterolateral and apical hypokinesia suggesting takotsubo cardiomyopathy (TCM). Atrioventricular block (AV) is rarely seen as initial presentation of TCM and has a prevalence of about 2.9%. AV block during early presentation of TCM poses a therapeutic dilemma with regard to the timing and the need to place a temporary or permanent pacemaker. The decision to place a permanent pacemaker may be on a case-by-case basis and more research is needed on formulating standardized recommendations in patients with TCM and conduction tissue abnormalities.

## 1. Introduction

Takotsubo cardiomyopathy is a distinct form of cardiomyopathy characterized by transient regional systolic dysfunction of the left ventricle (LV) and usually occurs secondary to severe emotional or physical stress [1, 2]. The presence of arrhythmias especially atrioventricular block used to be regarded an uncommon accompaniment of TCM but in recent year few reports of some association have emerged [3, 4]. Although TCM usually reverses completely within a few weeks, associated arrhythmias may be associated with poor prognosis [3–5].

The association of atrioventricular block with TCM may pose some therapeutic dilemma with respect to the need of a pacemaker and also the requirement for follow-up with a loop recorder [5].

We therefore present this case report with a view to elucidate the rare occurrence of atrioventricular block with TCM and possible interventions that may improve outcome in this patient population.

## 2. Case Description

**Figure 1:**
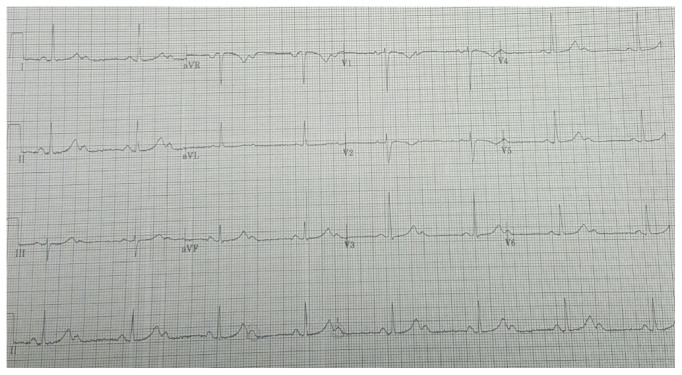
Initial EKG at the emergency department, showing 2nd-degree Mobitz type 2 atrioventricular (AV) block 2 : 1, heart rate 40 bpm, normal QRS voltage and complexes, T waves, and ST segments.

This is a 53-year-old African female patient who was brought in by emergency medical services (EMS) after the patient got trapped in the elevator. At that moment, she started feeling anxious and scared following which she activated EMS. EMS arrived at the scene after 20 minutes. During their evaluation, the patient stated she had retrosternal chest pain which was dull in quality, nonradiating, and 7/10 in severity. The chest pain had resolved at the time EMS arrived. At the time the patient arrived at our hospital, she was asymptomatic. Physical examination revealed a blood pressure of 146/77 mmHg, heart rate of 42 bpm, and respiratory rate of 16 per minute. Initial EKG as shown in Figure 1 demonstrated a 2nd-degree Mobitz type 2 atrial-ventricular (AV) block 2 : 1 with a heart rate of 40 bpm. Laboratory data were remarkable for troponin I 0.03 ng/ml (normal range is <0.02 ng/ml), pro-Brain Natriuretic Peptide 271 pg/ml, potassium 4.1 mmol/L, creatinine 0.8 mg/dl, and hemoglobin 12.9 g/dl. The patient had normal hepatic function test and thyroid stimulating hormone (3.49 mU/mL). Chest X-ray showed normal cardiac silhouette and no sign of fluid overload.

**Figure 2:**
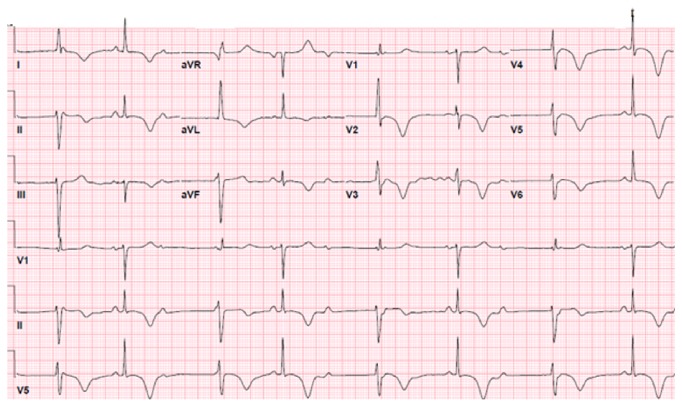
Electrocardiogram showing second-degree Mobitz type 2 AV block, 2 : 1 conduction, and marked T wave inversion on anterolateral leads suggesting ischemia.

Patient was admitted to coronary care unit (CCU) and a transcutaneous pacemaker was placed on demand. Labs were repeated 6 hours later after admission and troponin I trended up to 0.93 ng/ml, suggesting cardiac injury. Repeat EKG demonstrated T wave inversion in the anterolateral leads (Figure 2). Patient was started on heparin drip and loading doses of Plavix and aspirin were given. Our patient did not have *β*-blockers because of her presentation with bradycardia. Transthoracic 2D-echocardiogram revealed left ventricular ejection fraction (LVEF) of 45% as well as apical inferior, anterior, lateral, and septal hypokinesia.

**Figure 3:**
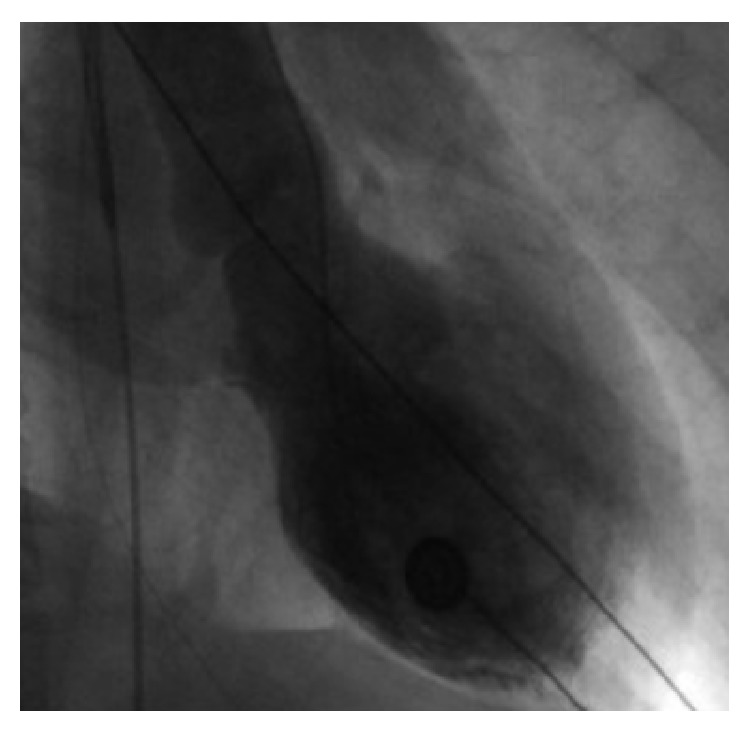
Left ventriculogram during end-systole phase, demonstrating basal segment contraction and anterolateral/apical hypokinesia.

Coronary angiogram was done in the view of new AV block, T wave inversion on EKG, and elevated troponin. Coronary angiogram demonstrated nonobstructive coronary artery disease. Ventriculogram demonstrated anterolateral and apical hypokinesia consistent with takotsubo cardiomyopathy (Figure 3). On further workup electrophysiology study revealed high-degree suprahisian AV block with intermittent 1 : 1 AV conduction, as well as 2 : 1. Recommendations were event monitor and follow-up.

Cardiac MRI was carried out just before discharge and showed normal LV ejection fraction (EF) of 66 and right ventricle EF of 66%. There was no evidence of myocardial scar, necrosis, or edema. LV apex appeared to be hypertrabeculated. Cardiac MRI findings were similar to those of the repeated transthoracic echocardiogram upon discharge which also showed LVEF of 60% with no wall motion abnormalities.

## 3. Discussion

TCM was first described in Japan by Sato in 1991 [1]. TCM is also referred to as apical ballooning syndrome, broken heart syndrome, or stress cardiomyopathy [6]. It is most commonly seen in postmenopausal women and frequently precipitated by a stressful event as seen in our patient [4]. TCM is characterized by transient LV dysfunction with typical distinct wall motion abnormalities of the apical and/or midventricular or basal segments [7]. The pathogenesis is not well understood but catecholamine excess has been postulated as being central to the pathogenesis of TCM [1]. Some researchers have found that circulating levels of epinephrine and norepinephrine may be 2 to 3 times higher in TCM than in patients with acute myocardial infarction [1].

Although the clinical presentation simulates that of an acute myocardial infarction, coronary arteriography typically shows no obstructive lesions [1]. The most common clinical symptoms among patients with TCM are chest pain and dyspnea but other rare presentations may occur [4, 8].

Arrhythmias have been described as an unusual complication of TCM and may include ventricular arrhythmia, atrial fibrillation, torsade de pointes, and conduction tissue dysfunction [8, 9]. Atrioventricular block (AV) is also a rarer association with TCM and has a prevalence of about 2.9% [9]. Few reports suggest that AV block may be commoner in elder women who may have aging as a contributor to AV dysfunction unlike what was seen in our 56-year-old patient. The association of complete AV block and TCM remains a conundrum because the damaged apical ventricle is some distance away from the conduction pathways in the atrium, bundle of His, atrioventricular node, and intrahisian region [3]. As a result, some authors suggest that diffuse spasms in small branches of the coronary arteries may be responsible for both myocardial ischemia and atrioventricular conduction disturbances [4].

It is interesting to note that many cases of high-degree AV block coexisting with TCM had persisted even after recovery of myocardial function necessitating permanent cardiac pacemaker implantation [4]. It is possible that the recovery of the cardiac conduction system is prolonged and lags that of the myocardial tissue recovery in patients with TCM [4, 8]. Nault et al. demonstrated, in an electrophysiological study, lack of resolution of a high-degree AV block 1 year after TCM which eventually resolved 2 years later [4].

Therefore, AV block during early presentation of TCM poses a therapeutic dilemma with regard to the timing and the need to place a temporary or permanent pacemaker. The decision to place a permanent pacemaker may be on a case-by-case basis because of the ongoing debate in view of the reversible nature of TCM and attendant complications of permanent pacemaker devices such as infection and inappropriate shocks [7]. The placement of a temporary pacemaker in our patient appears to have sufficed and she did not require permanent pacemaker in the long run. Following electrophysiology, our patient was noted to have high-degree suprahisian block. Other authors may place a permanent pacemaker if AV block persists and or if infrahisian block is seen during electrophysiology studies [3]. Shanmugasundaram et al. [4] also reported placing a permanent pacemaker after 18 days of persistent AV block while the patient was on a temporary pacemaker during recovery from TCM.

Caution should also be taken with regard to use of *β*-blockers which has been described by some authors as the mainstay of therapy among patients with TCM because of the strong link with catecholamine excess [7].

Dangerous arrhythmias such as ventricular tachycardia and complete AV block are possibilities in patients with TCM when features of conduction tissue disease are present. Placing and event loop recorder as in our case might have been a wise option as missing sinister arrhythmias may be lethal during the recovery period in some patients with TCM [5].

## 4. Conclusion

Takotsubo cardiomyopathy is rare disease and its coexistence with high-degree atrioventricular block is rarer. The prognosis of TCM is good, but extra care should be taken when there is coexistent conductive tissue disease as the need for permanent pacemakers may arise and fatality rate may also be high due to occurrence of malignant arrhythmias. More research is needed on formulating standardized recommendations in patients with TCM and conduction tissue abnormalities.
